# Transvenous pulmonary chemoembolization and microwave ablation for lung metastases from breast cancer: a propensity score matching analysis

**DOI:** 10.1007/s11547-025-01966-4

**Published:** 2025-03-18

**Authors:** Hamzah Adwan, Lars Hammann, John Bielfeldt, Sven Becker, Thomas J. Vogl

**Affiliations:** 1 Clinic for Radiology and Nuclear Medicine, University Hospital Frankfurt, Goethe University, Theodor-Stern-Kai 7, 60590 Frankfurt Am Main, Germany; 2 Department of Gynecology and Obstetrics, University Hospital Frankfurt, Goethe University, Theodor-Stern-Kai 7, 60590 Frankfurt Am Main, Germany

**Keywords:** Transvenous pulmonary chemoembolization, Microwave ablation, Breast cancer lung metastases

## Abstract

**Purpose:**

To compare the outcomes of patients with pulmonary metastases from breast cancer, who were treated with transvenous pulmonary chemoembolization (TPCE) and consecutive microwave ablation (MWA) with patients treated by TPCE alone.

**Material and methods:**

This retrospective single-center study included patients with unresectable and/or non-responsive to systemic chemotherapy pulmonary metastases originating from breast cancer, treated by TPCE followed by MWA, in case of adequate response to TPCE, or by TPCE alone. The groups of patients were balanced using propensity score matching (PSM).

**Results:**

A total of 97 patients met the inclusion criteria for this study. After PSM, 23 patients were included in the combination therapy group (Group 1) and 42 patients were included in the monotherapy group (Group 2). The median overall survival (OS) time was 33.6 months for Group 1 with a 2-year OS rate of 62%, and 20.2 months for Group 2 with a 2-year OS rate of 43%. There was no significant difference between the two groups regarding OS (*p* value: 0.429). The rate of progressive/recurrent disease was 17.4% (4/23) in Group 1 and 23.8% (10/42) in Group 2 (*p* value: 0.754). The number of metastases was the only significant factor for OS in all patients after PSM (*p* value: 0.032, HR: 1.016, 95% CI 1.001–1.031).

**Conclusion:**

TPCE is an effective potential treatment for lung metastases of breast cancer, which can be performed alone or combined with MWA. Patients who responded to TPCE and received subsequent MWA demonstrated non-significant better OS and local tumor control.

## Introduction

Breast cancer is the most common type of cancer and is responsible for the most deaths from cancer among women globally [1]. Breast cancer is prone to develop metastases, which are the primary cause of mortality associated with the disease [2]. Chemotherapy is an essential pillar in the management of advanced and metastatic breast cancer [3]. However, chemotherapy is associated with several serious systemic side effects such as neutropenia and neuropathy, among others [3].

Local minimally invasive therapies play a vital role in the treatment of cancer. Especially in the treatment of primary cancers [4], but also in the treatment of metastases in various locations, such as the liver or lung [5, 6]. These minimally invasive treatments include local ablative procedures, such as cryoablation (CA) [7], radiofrequency ablation (RFA) [8], laser-induced thermotherapy (LITT) [9], and microwave ablation (MWA) [10].

These techniques utilize the application of thermal energy, in the case of MWA, electromagnetic energy transmitted via microwaves, to heat the tissue surrounding the applicator [11]. The application of heat leads to cell damage and tumor cell necrosis [10].

The efficacy of these procedures varies with the dimensions of the targeted lesions, with better results in lesions with smaller diameter [12]. Therefore, larger lesions may require downsizing prior to ablative treatment, in order to ensure a complete ablation and a potentially curative treatment outcome [13]. Downsizing of index lesions can be achieved through the utilization of different therapies such as systemic chemotherapy or more targeted approaches. These include the local intravascular application of chemotherapeutic drugs [14]. In this study transvenous pulmonary chemoembolization (TPCE) was selected as treatment for pulmonary metastases of breast cancer. This enables the administration of chemotherapeutic agents followed by embolic agents directly into the tumor-feeding vessels, in order to initiate a volume reduction of the target lesion, as shown before in other studies [15, 16]. Another benefit of locally administering chemotherapeutic agents is the ability to achieve higher concentrations in the target area, without raising the risk of systemic side effects [17, 18]. The subsequent application of embolic agents after the administration of chemotherapeutic drugs induces a blood stasis in the tumor-feeding vessels, preventing the elimination of the chemotherapeutic drugs and, moreover, preventing blood flow to the tumor, therefore, inducing ischemia and cell necrosis [19, 20].

The efficacy of transvascular approaches in the treatment of intrapulmonary malignancies has been demonstrated previously, particularly for the treatment of primary lung cancer [21]. Xu et al. could show that the combination of drug-eluting bead bronchial arterial chemoembolization (DEB-BACE) and MWA in the treatment of non-small cell lung cancer (NSCLC) can result in significantly longer progression-free survival times and can achieve disease control rates of up to 85.7% compared to DEB-BACE alone [22]. However, studies evaluating the application of TPCE or BACE for lung metastases of non-primary lung cancers remain scarce. The existing data primarily consist of retrospective studies on the use of TPCE in primary lung tumors as well as in lung metastases of  different primaries [15, 23]. Accordingly, this study assesses the treatment of patients with lung metastases explicitly from breast cancer who were treated with TPCE. In cases where an adequate response to TPCE was registered, subsequent treatment with MWA was employed to ablate the remaining index metastasis.

## Material and methods

This single-center study was approved by our Internal Review Board (IRB) and conducted in conformity to the ethics guidelines of the Declaration of Helsinki and its amendments. Due to the retrospective design, an informed consent to study participation was waived.

This study analyzed patients with unresectable, and/or non-systemic chemotherapy-responsive pulmonary metastases of breast cancer, who were treated at the Clinic for Radiology and Nuclear Medicine of our university hospital. The patients were divided into two groups depending on their response to TPCE. All patients initially received repeated TPCE at intervals of four to six weeks, with the objective of devascularizing the tumors and decreasing their size and number. Accordingly, the response to TPCE was assessed using cross-sectional imaging. Patients who sufficiently responded to TPCE were subsequently treated with MWA (Group 1). Patients who did not adequately respond to TPCE did not receive a subsequent treatment by ablation and were solely treated with TPCE (Group 2).

### Inclusion and exclusion criteria

Patient characteristics included in an institutional dedicated database were screened regarding eligibility for inclusion in this study. Therefore, the following inclusion criteria were formulated: (1) pulmonary metastases of breast cancer, which are for any reason unresectable and/or unresponsive to systemic chemotherapy, (2) metastases were treated by repeated TPCE and optionally received a subsequent treatment via MWA, and (3) had available sufficient follow-up MRI and CT scans to evaluate treatment response. Patients with concomitant bone metastases were included if those were clinically and radiologically stable.

Patients were excluded from the study if they met any of the following exclusion criteria: (1) evidence of existing pulmonary arteriovenous shunts, (2) notes on the presence of central or peripheral pulmonary embolism, (3) respiratory failure, (4) multiple primary tumors, (5) liver or brain metastases (6) pleural carcinomatosis (7) insufficient coagulation (platelet count < 50,000 per μL/ international normalized ratio [INR] > 1.5), (8) leukopenia or massive anemia, (9) poor general condition, (10) intolerance toward contrast medium, or (11) cardiac failure.

### Patient population and study design

**Fig. 1:**
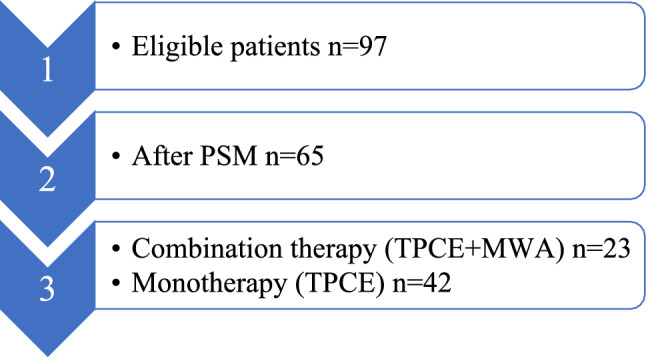
Methodological diagram showing the study design

A total of 97 patients (95 females and 2 males; median age: 59 years), who had undergone TPCE treatment, with or without subsequent MWA treatment, met the inclusion criteria of our study. After propensity score matching (PSM), 65 patients were divided into separate groups (Fig. 1). A total of 23 female patients with a median age of 58 years were included in the combination therapy group (Group 1), while 42 female patients with a median age of 57 years were included in the monotherapy group (Group 2). The details of the patients’ characteristics and their metastases are provided in Table 1.

**Table 1:** Patients’ and metastases’ characteristics

	Group 1	Group 2
Number of patients (% of total)	23 (35.4)	42 (64.6)
Female *n* (%)	23 (100)	42 (100)
Male *n* (%)	0 (0)	0 (0)
Median age in years	58	57
Patients with 5 metastases or less (%)	13 (56.5)	18 (42.9)
Patients with more than 5 metastases (%)	10 (43.5)	24 (57.1)
Patients with reference metastasis of ≤ 2 cm (%)	15 (65.2)	26 (61.9)
Patients with reference metastasis of > 2 cm (%)	8 (34.8)	16 (30.1)
Patients treated by less than ten sessions of TPCE (%)	8 (34.8)	5 (11.9)
Patients treated by ten sessions of TPCE or more (%)	15 (65.2)	37 (88.1)

### Combination therapy group (Group 1)

This group consisted of 23 patients, including only women with a mean age of 57.9 ± 9.5 years and a median age of 58 years (range: 41–71 years), who were primarily treated with repetitive TPCE, and after reducing the size and number of metastases, received MWA.

The patients initially had a mean number of metastases of 16.5 ± 27.2 and received a mean of 9.6 ± 6.9 sessions of TPCE at four- to six-week intervals. The patients were afterwards treated by at least one session of MWA.

### Monotherapy group (Group 2)

A total of 42 patients could be included in the monotherapy group and received only TPCE. The patient group consisted entirely of women with a mean age of 57.8 ± 9 years; median 57 years (range: 39–74 years).

Patients had predominant lung metastases with a mean metastatic count of 15.1 ± 21.9.

This group underwent an average of 5.2 ± 3.8 sessions of TPCE at intervals averaging four to six weeks during the course of therapy.

### Interventional procedures

The protocols for TPCE and MWA procedures were similar to our previous study published by our institute on the treatment of lung metastases of colorectal cancer [24]. Informed consent was acquired from each patient before each therapy session. The following important laboratory parameters were evaluated prior to each therapy session: hemoglobin, creatinine, INR and platelet counts.

To initiate the procedure the selected incision site in the patient’s groin was infiltrated with 1% mepivacaine, thereby achieving local anesthesia. Following, a sheath was inserted via femoral venous access and a catheter was introduced. For TPCE, various catheters were utilized, such as pigtail, headhunter and cobra catheters, among others. To identify and evaluate the relevant tumor-supplying vessels, a pulmonary angiographic review was conducted. Subsequently, the pulmonary artery branches supplying the metastases were selectively or super-selectively catheterized, and a combination of several chemotherapeutic drugs was injected.

An individual combination and dosage, determined by a multidisciplinary tumor board, of mitomycin C 8 mg/m^2^ (mito-medac®, Medac), cisplatin 35 mg/m^2^ (Cisplatin Accord, Accord Healthcare Limited), and either gemcitabine 800 mg/m^2^ (Gemcitabin HEXAL®, Hexal AG) or irinotecan 100 mg/m^2^ (Irinotecan Aurobindo®, PUREN Pharma GmbH) was mostly utilized. To achieve a subsequent blood stasis in the tumor-supplying vessels, up to 10 mL of Lipiodol® (Guerbert, Germany) and 200–450 mg microspheres (EmboCept, PharmaCept GmbH, Germany) were gradually administered under fluoroscopy.

Important laboratory parameters were once again evaluated before each MWA treatment. All MWA treatments were performed percutaneously under unenhanced computed tomography (CT)-guidance (Somatom Definition AS, Siemens). At first, the targeted metastasis was localized using a planning CT scan and a sufficient incision trajectory was determined. Subsequently, local anesthesia was applied under sterile conditions and the microwave antenna was cautiously positioned in the center of the targeted metastasis. After the completion of the ablation procedure the antenna was removed, and the incision track was sealed.

### Follow-up

Radiological follow-ups were conducted both before and after treatment using CT (Siemens Healthcare, Erlangen, Germany) and magnetic resonance imaging (MRI) (Siemens Healthcare, Erlangen, Germany), in order to detect treatment-related complications and monitor the therapy response. The diameter of each reference lesion was measured before starting the treatment. For Group 1, reoccurring tumor growths were categorized as either local tumor progression (LTP) or intrapulmonary distant recurrence (IDR) as described by Zuo et al. [25]. Furthermore, in Group 2 the reference lesions were analyzed in accordance with the revised Response Evaluation Criteria in Solid Tumors (RECIST 1.1) [26].

### Statistical analysis

The statistical analyses and calculations were performed with SPSS® (Statistical Package for the Social Sciences, GradPack 27.0, IBM, Armonk, USA) and R (R Foundation for Statistical Computing, Vienna, Austria). The package ‚MatchIt’ was used. For each patient, a number of parameters were documented, including age, sex, the number and diameter of intrapulmonary metastases. The patients in groups 1 and 2 were balanced using PSM in order to reduce potential bias. The variables considered in the PSM were age, sex, number of metastases as well as diameter of reference metastasis. Furthermore, therapy response and survival times were documented. Overall survival (OS) was calculated from the date of treatment until the date of last contact or death from any cause. For the calculation of survival rates the Kaplan–Meier method was used. The survival of the groups was compared using the log-rank test. We used the Fisher's Exact Test to compare nominal variables. Prognostic factors for OS were identified through Cox regression analysis. A *p* value of 0.05 or less was deemed statistically significant.

## Results

### Local tumor response

**Fig. 2:**
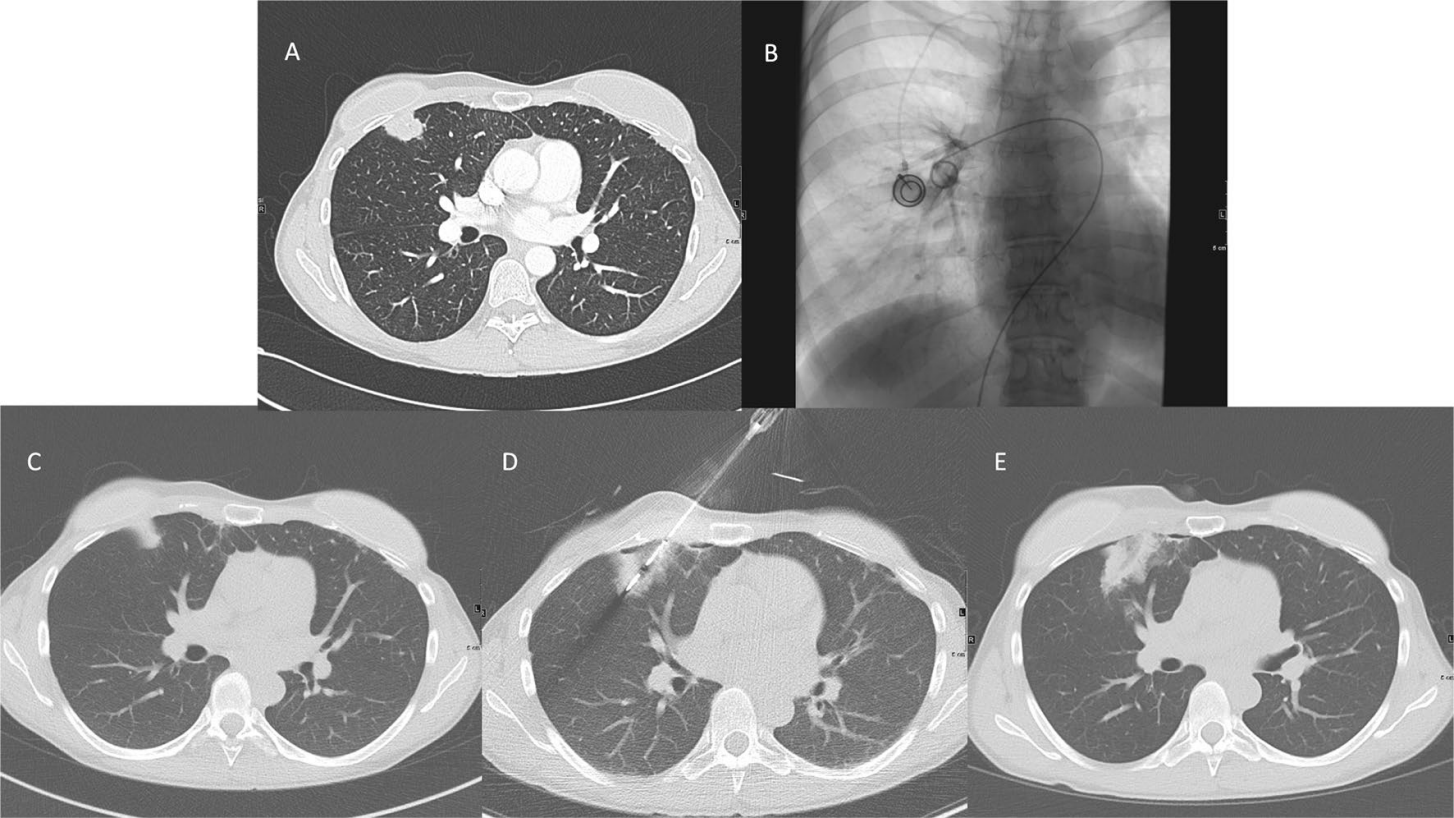
A 41-year-old woman with lung metastases from breast cancer treated by repetitive TPCE followed by MWA. **A** Initial CT scan shows a metastasis in the right lung. **B** During TPCE. **C** CT scan after four sessions of TPCE shows size reduction of the metastasis. **D** CT scan during MWA therapy. **E** CT scan one day after MWA

In Group 1, a total of four patients at a rate of 17.4% (4/23) developed IDR. There were no cases of LTP in Group 1. In Group 2, partial response (PR) was achieved in 9.5% of cases (4/42), while stable disease (SD) was achieved in 66.7% of cases (28/42). Therefore, the rate of local disease control (PR + SD) was 76.2% (32/42). Progressive disease (PD) occurred in 23.8% (10/42) of the patients in Group 2. The difference between both groups regarding progressive/recurrent disease was not significant (*p* value: 0.754). Figure 2 displays the case of a patient included in Group 1 who received a combination of TPCE and MWA.

### Survival analysis

**Fig. 3:**
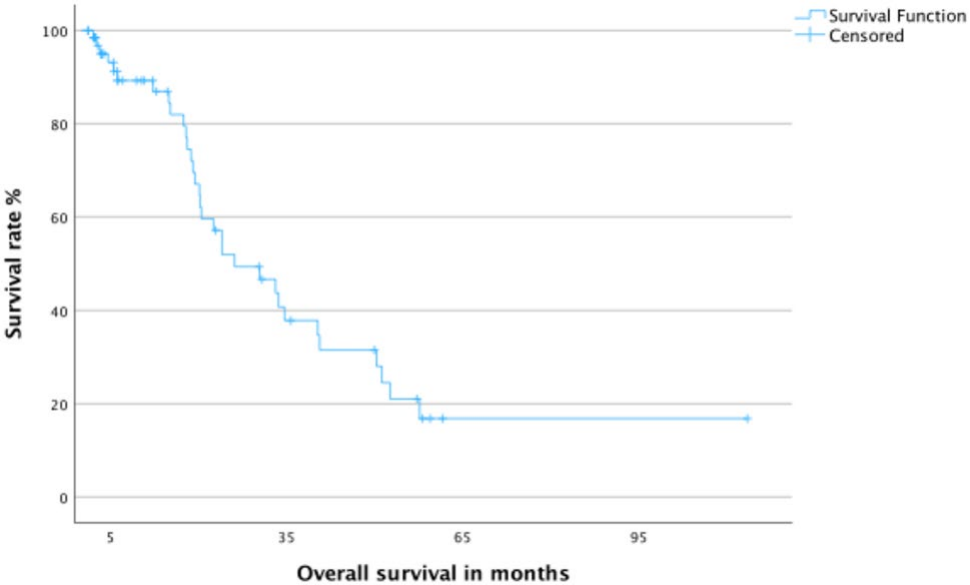
Kaplan–Meier curve for overall survival for all patients

**Fig. 4:**
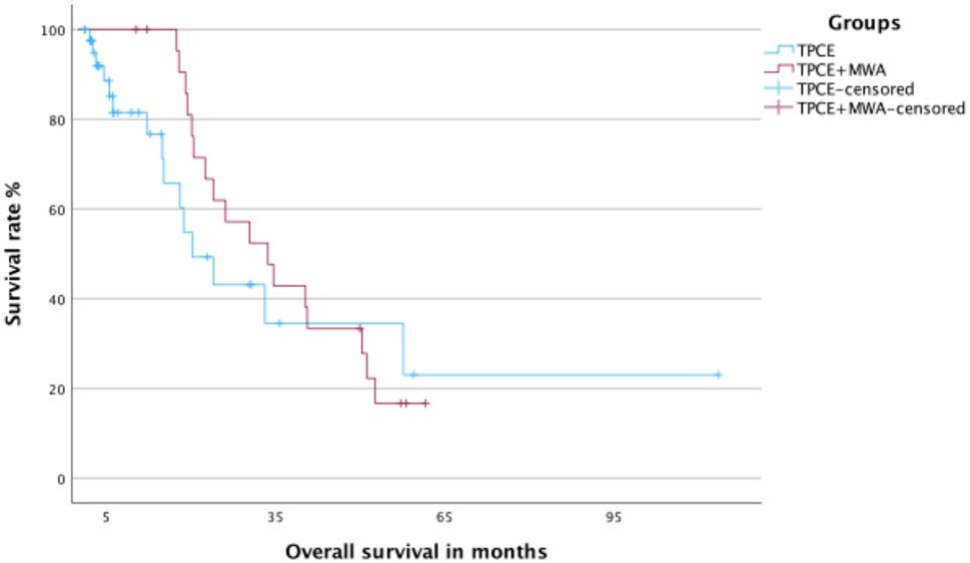
Kaplan–Meier curves for overall survival for group 1 (TPCE + MWA) and group 2 (TPCE)

The median OS time for all 65 patients was 26.12 months (95% CI 14–38) with a 2-year OS rate of 51.9%. For group 1, the median OS time was 33.6 months (95% CI 20.8–46.3) with a 2-year OS rate of 61.9%. Group 2 had an OS time of 20.2 months (95% CI 12.6–27.8) with a 2-year OS rate of 43.1%. The difference between the two groups was not statistically significant (*p* value: 0.429). The Kaplan–Meier curves for OS for all patients and for both main groups are shown in Figs. 3 and 4, respectively.

Furthermore, the 65 patients were divided by the number and size of existing metastases. For patients with five or less metastases the median OS time was 51.2 months (95% CI 33.5–68.8), compared to 20.1 months (95% CI 17.9–22.3) for patients with more than five metastases. Hereby, the difference was statistically significant (*p* value: 0.0006). The median OS time for patients with a diameter of the reference metastasis of ≤ 2 cm was prolonged at 30.3 months (95% CI 17.8–42.9) compared to patients with diameter of reference metastasis of more than  2 cm at 22.5 months (95% CI 3–42). However, the difference was not statistically significant (*p* value: 0.125). The survival outcomes are summarized in Table 2.

**Table 2:** Survival outcome

Parameter	Value	*p* value
Median OS in months (95%CI) total	26.12 (14–38)	0.429
Group 1	33.6 (20.8–46.3)	
Group 2	20.2 (12.6–27.8)	
2-year OS rate in %		
Group 1	61.9	
Group 2	43.1	
Median OS by number of metastasis (months)		0.0006
Five or less	51.2 (33.5–68.8)	
More than five	20.1 (17.9 -22.3)	
Median OS by diameter of references metastasis (months)		0.125
≤ 2 cm	30.3 (17.8–42.9)	
> 2 cm	22.5 (3–42)	

### Prognostic factors for overall survival in all patients after PSM

The age of the patients (*p* value: 0.561, HR: 0.988, 95% CI 0.948–1.029), the number of performed sessions of TPCE (*p* value: 0.247, HR: 0.962, 95% CI 0.901–1.027) as well as diameter of reference metastasis (*p* value: 0.234, HR: 1.205, 95% CI 0.886–1.639) were not significant factors for OS. The number of metastases (*p* value: 0.032, HR: 1.016, 95% CI 1.001–1.031) was the sole significant factor for OS. The prognostic factors for OS in all 65 patients are summarized in Table 3.

**Table 3:** Prognostic factors

	Hazard Ratio	p-value	95% CI
Patient age	0.988	0.561	0.948–1.029
Number of TPCE sessions	0.962	0.247	0.901–1.027
Diameter of reference metastasis	1.205	0.234	0.886–1.639
Number of metastases	1.016	0.032	1.001–1.031

## Discussion

Breast cancer should be seen as a systemic disease, in which the metastases are a major reason for mortality among patients [27]. Therefore, patients with breast cancer should be treated using a multimodal therapy concept. This study discussed patients’ survival and tumor response after local therapy with TPCE alone or combined with subsequent MWA for patients with unresectable and/or non-systemic chemotherapy-responsive lung metastases of breast cancer. To enhance comparability between the two groups, a PSM was conducted for the purpose of this study.

The evidence on this topic remains limited, with only a small number of studies for different tumor types, such as primary lung cancer, and minimal survival data available [21, 22]. Nevertheless, our findings suggest that the combination of these two treatments may lead to improved survival, although the observed effect failed to reach statistical significance. Similar results could be shown in the case of pulmonary metastases of colorectal cancer, with a median survival of 29 months for the combination of TPCE and MWA and 15 months for the monotherapy alone [24].

Thus, the combination of MWA and TPCE may provide a beneficial therapy option for local tumor control for patients who adequately respond to TPCE, with the potential to prolong OS.

A beneficial outcome after the combination of vascular embolization and subsequent ablation has been shown before, although these have been primarily observed in the treatment of hepatic malignancies such as hepatocellular carcinoma (HCC) [28, 29]. Though it has also been shown, for instance by Wang et al., that hepatic metastases of breast cancer can be effectively treated via transarterial chemoembolization and RFA, and this may lead to an improved progression-free survival and OS [30].

Moreover, metastases of breast cancer can be treated by thermal ablation including pulmonary metastases, which may lead to favorable tumor control rates [31]. In a study conducted by Meng et al., patients with lung metastases originating from breast cancer were treated via MWA, however the ablations were performed without previous downsizing of the tumors through TPCE [32]. The 1- and 3-year OS rates were 96.9% and 53.3%, respectively, with a median OS time of 36 months. Therefore, the results by Meng et al. are similar to those of our study, despite the discrepancy in reported LTP rate of 10.9% compared to 0% in our study. The TPCE in our study may have an impact on local tumor response, potentially facilitating a complete ablation process through smaller tumors.

Of the patients treated with TPCE alone, a local disease control could be achieved in 76.2% (32/42) of patients. Hori et al. demonstrated that transarterial chemotherapy followed by chemoembolization is an effective treatment for locally recurrent breast cancer, ensuring a 7.2% 6-months complete response rate and 67.8% partial response rate [33]. These results support the application of chemoembolization for breast cancer therapy, although our study focused on its metastases. Nonetheless, TPCE may be applied as a palliative treatment option for patients lacking a curative treatment option, with the objective of prolonging survival.

In addition, we could show that the sole significant prognostic factor for survival is the total number of metastases in the lung. Also, the median OS for patients with five or less metastases was significantly longer than the median OS of patients with more than five metastases. These findings can be supported by a study conducted by Ferguson et al., who could also show that patients with three or less metastases, though originating from colorectal cancer, had a longer median survival of 33.7 months, compared to 26.3 months for patients with four or more metastases [34]. This underscores the necessity of effectively treating and reducing the burden of metastases to potentially prolong survival.

It must be highlighted that despite all efforts, there are some limitations to our study. Firstly, it is a retrospective data analysis conducted at a single institution. For an optimal evaluation, a prospective, randomized trial is required. Secondly, the number of patients was limited, particularly in the combination therapy group. However, we tried to enhance comparability between the groups through PSM. Lastly, some important factors, which may have an impact on survival of the patients, such as the histological type of breast cancer, could not be considered due to the retrospective nature of study.

## Conclusion

TPCE is a potential treatment option for patients with unresectable and/or unresponsive to systemic chemotherapy pulmonary metastases from breast cancer. If patients adequately respond to TPCE treatment, it may also be combined with MWA to improve local tumor response and OS. Hereby, the number of metastases can be used as a prognostic factor for the patients’ survival. However, further prospective randomized studies are required to verify these findings.
